# The Association of the Alumni and Officers of the Medical Department of the University of Buffalo

**Published:** 1876-01

**Authors:** 


### The Alumni Association of the Medical Department of the University of Buffalo.

The Executive Committee of the Association of the Alumni and officers of the Medical Department of the University of Buffalo have issued the following circular to which we take great pleasure in calling the attention of those of our readers who are alumni of the Buffalo Medical College:

*Association of the Alumni and Officers of the Medical Department of the University of Buffalo.*

OFFICE OF THE EXECUTIVE COMMITTEE,
Buffalo, N. Y., Jan. 20, 1876.

DEAR DOCTOR:

The Second Annual Meeting of this Association will be held on Wednesday, February 23d, 1876, at ten o'clock A. M., in the College building, corner of Main and Virginia streets. In the evening at seven and a half o'clock the commencement exercises of the College will be held at St. James Hall. The address to the graduating class will be delivered by M. B. Anderson, LL. D., President of the Rochester University, following which the annual address to the Alumni will be delivered by Sanford B. Hunt, M. D., of Newark, N. J., formerly Professor of Anatomy in the Medical department of the University of Buffalo. At the conclusion of the exercises at the Hall a supper will be served at the Tifft House.

The order of business at the meeting at the College will embrace the following: An address by the President of the Association, Dr. W. W. Jones, (49) of Toledo, O., Essay by Dr. C. H. Richmond, (60) of Livonia Station, N. Y., Subject, Questions Relating to Sanitary Science, A Paper by Dr. S. H. Benton, (70) of Oil City, Pa., on Hygiene and Sanitary Reform, and a poem by Dr. F. Bradnack, (71) of New York City.

The pronounced success of the inaugural meeting of this Association in February last, and the well known ability of the gentlemen who are to address the Association and present papers for discussion at the coming meeting, warrant the committee in assuring you that your attendance, which is strongly urged, will be both pleasant and profitable.

The committee desires to state that the character of the papers which will be presented will confine the discussion to a subject which is daily assuming more importance, viz.: Sanitary Science, and they therefore suggest that you come prepared to discuss that topic. In their preparations for the supper, etc., it is important that the committee should know how many will be present, you will therefore confer a favor by communicating with us at an early day in reference to your attendance.

Very sincerely yours,

W. W. POTTER, M. D., Chairman,
E. N. BRUSH, M. D., Secretary,
A. H. BRIGGS, M. D.,
W. W. JONES, M. D., President and member *ex officio*,
M. G. POTTER, M. D., Dean of College, and member *ex officio*.

Please address all communications to Dr. E. N. Brush, Secretary of the Committee, No, 8, S. Division St., Buffalo, N. Y.

